# Public health the leading force of the Indonesian response to the HIV/AIDS crisis among people who inject drugs

**DOI:** 10.1186/1477-7517-4-9

**Published:** 2007-02-17

**Authors:** Fabio Mesquita, Inang Winarso, Ingrid I Atmosukarto, Bambang Eka, Laura Nevendorff, Amala Rahmah, Patri Handoyo, Priscillia Anastasia, Rosi Angela

**Affiliations:** 1 Indonesia HIV/AIDS Prevention and Care Project, Jakarta, Indonesia; 2 Indonesian National AIDS Commission, Jakarta, Indonesia; 3 Indonesia HIV/AIDS Prevention and Care Project, Bandung, Indonesia; 4 Indonesia HIV/AIDS Prevention and Care Project, Bali, Indonesia

## Abstract

**Issue:**

Indonesia has an explosive HIV/AIDS epidemic starting from the beginning of this century, and it is in process to build its response. Reported AIDS cases doubled from 2003 – 2004, and approximately 54% of these cases are in people who inject drugs.

**Setting:**

Indonesia is the 4^th ^largest country in population in the world, a predominantly Muslim country with strong views on drug users and people living with HIV/AIDS. Globally speaking, Indonesia has one of the most explosive epidemics in recent years.

**The project:**

IHPCP (Indonesia HIV/AIDS Prevention and Care Project) is a joint support project (primarily AusAID-based) that works in partnership with the Government of Indonesia. IHPCP has been a key player of in the country's response, particularly pioneering NSP; stimulating and supporting methadone programs, and being key in promoting ARV for people who currently inject drugs. The project works via both the public health system and NGOs.

**Outcomes:**

It is still early to measure the impact of current interventions; however, this paper describes the current status of Indonesia's response to the HIV/AIDS crisis among people who inject drugs, and analyses future challenges of the epidemic in Indonesia.

## I. Background

According to the last UNAIDS report on the global HIV/AIDS epidemic, the core expansion of the HIV/AIDS epidemic (absolute number of cases reported) is currently based on injecting drug use in Asia and Eastern Europe [1]. India recently achieved the biggest number of reported AIDS cases of any country globally, however the two major epidemics in Asia – mainly driven by injecting drug use – are in China and Indonesia. This paper reports the current situation in Indonesia by the end of 2006, and how the national response to this crisis is being built by the Indonesian government, civil society and external partners.

Indonesia is a country of approximately 17,000 islands, with the fourth largest population in the world. It is a predominantly Muslim country with strong views on drug users, sex (use of condoms) and people living with HIV/AIDS.

After 32 years dominated by a military dictatorship, the democratization process is very recent, having started in 1998. As part of this process, decentralization of power and budgets, and consequently decentralization of the responsibilities on public policies and governance, has a clear impact on the public health system. As time passes, cities, districts and provinces are addressing the alignment of responsibilities in public health matters. The decentralization of the response to the HIV/AIDS epidemic is an ongoing process with increasing responsibilities shared among different levels of government.

The epidemic of HIV/AIDS in Indonesia reported its first case of AIDS in 1987. The first reported AIDS case among people who inject drugs (IDU) was in 1995. Since then, IDUs have constituted a major component of the country's epidemic [2]. According to the Centre for Disease Control (CDC) of the Ministry of Health of Indonesia, reported AIDS cases doubled from 2003 – 2004, and approximately 80% of the new cases in the last two years are among people who inject drugs. Cumulatively, transmission of HIV related to the use of injectable drugs accounts for 54% of the total AIDS cases in the country [3]. National estimates indicate that the number of people living with HIV/AIDS ranges from 165,000 to 216,000 [4]. Widespread, free access to an HIV test is a recent phenomenon; the logistics of the system is still being worked out. Available data is not accurate; there is as well the need to increase quality of data collection and flux of the information.

Currently, there are many bodies of the Government playing a role in the control of the HIV/AIDS epidemic, primarily the KPA or the National Commission on AIDS, which has been attached to the Presidential Cabinet from July 2006. With a recently empowered strong leadership, KPA is in the process of recruitment to build their internal team with some of the best staff in the field of HIV/AIDS in the country and has a very promising role in response leadership. KPA is not involved in policy implementation, but rather responsible for formulating policies, and works mainly with international sources – centred on DFID, the British Cooperation – via partnership funds, which are administrated by UNDP. UNAIDS is the multilateral organization that provides technical support to KPA.

The Ministry of Health is responsible for implementating the response to the HIV/AIDS epidemic, comprised of four departments. The Pharmacy Department is responsible for all medications. The Centre for Diseases Control includes the National AIDS Program which is responsible for program development, building local human resources and for all matters related to epidemiology. The Department of Medical Services runs all the hospitals, the Drug Program (including methadone clinics), and all laboratories. Lastly, the Community Health Department is responsible for the Community Public Health Centres (Puskesmas) programs. It has been somewhat difficult to integrate all departments in one coordinated implementation of the HIV/HIV/AIDS response. WHO is the multilateral organization that works closely with the Ministry of Health to assist the Indonesian national response.

At the national level in the harm reduction field is the National Narcotic Board (BNN), which is attached to the National Police. This body is also responsible for narcotic demand and supply reduction, their primary focus. Also related to this effort is the Ministry of Justice and Human Rights, which runs prisons in the country and is responsible for every intervention inside the prison system.

In addition to the Indonesian government sectors, the international community is involved in the country's HIV/AIDS response. Indonesia received $64 million US from the fourth round of the Global Fund with a project whose scope contains what is required to confront the epidemic, including a detailed cost study build in the WHO model (Costing Guidelines for HIV/AIDS Intervention Strategies). The Ministry of Health, through the Centre for Disease Control, leads the implementation of the Global Fund project. Unfortunately in Indonesia, administration of the Global Fund sources has led to a "D" classification, with results below expectations [5]. National and international experts in the country agree that the lack of good reporting process could be influential in establishing this classification. In addition to the Global Fund, DFID, USAID, AusAID and KFW are working in Indonesia in the field of HIV/AIDS. WHO, UNAIDS and recently UNODC, among other UN agencies, also have a strong influence on the response thus far. Other international agencies have minor influence in specific aspects of the response in Indonesia.

In addition to the efforts from the Indonesian national government and international partners, there are local responses organized in several provinces and cities, in conjunction with the decentralization process already mentioned. Commitments are different based on the specific local history and importance of the epidemic, as well as the political climate of the various local governments. To complete this complex framework, Non-Governmental Organizations (NGOs) were involved at the onset and are still crucial in the Indonesian response to the HIV/AIDS epidemic.

With permeable borders in its 17,000 islands, geographically close to the Golden Triangle, and as well not greatly distant from Afghanistan, since the late 90s, Indonesia has become a great market for heroin, and currently also a rising market for amphetamines. In its 2005 report, the National Narcotics Board indicated that there are 3.2 million drug users in Indonesia of which 25% are heavily addicted and injecting drugs [6]. Still, according to BNN, the trends of drug use are measured by drug treatment admissions in hospitals, admissions in rehabilitation centres, drug seizures, prisons for drug offences, and injecting drug users reported by the Ministry of Health as AIDS cases. According to the sum total of this information, marijuana is the number one drug of abuse, followed by heroin, amphetamine type stimulants (ATS), hashish and cocaine. There is an increased availability of night drugs such as ecstasy also available in Indonesia. Poly-drug use, sedative hypnotic drugs and drugs of inhalation are also being reported. As already mentioned, BNN manages demand reduction, which for Indonesia includes: *"prevention (family based, school based, community based and workplace based) treatment and rehabilitation activities in both public, NGO, and private facilities, employing various modalities. Supply Reduction Strategies are implemented through more intensive eradication of cannabis cultivation, intensive investigations and raids of clandestine manufacturers and applying strict airport and seaport interdictions" *[7]. Burnet Institute's Centre for Harm Reduction in collaboration with the Turning Point Alcohol and Drug Centre conducted a recent situational analysis in Indonesia (as well as other countries in Asia) on behalf of the Australian National Council on Drugs and found similar information on drugs, drug supply and demand reduction [8]. Under the Indonesian legislation, the use of drug is criminal (this is also true of possession) and trafficking is punishable by the death penalty. The strict criminalization of drug use behaviours has made it difficult to reach injecting drug users for health care services and harm reduction programs.

At the early stage of the epidemic among drug users in the late 90's, the response was dominated by NGOs supported by international aid agencies such as USAID and AUSAID [9]. Local governments were not showing the commitment needed for the response while the central government was just beginning to get more exposure to the problem and to harm reduction approaches.

Regarding harm reduction, the first recorded NGO organizing harm reduction services was Yayasan Hati-hati (Bali-based) in 1998. Since then, more organizations developed in many parts of the country, the majority founded after the beginning of the 21^st ^century. All of these organizations are made up of people with previous experience in the drug field (the majority former drug users) to address the AIDS epidemic among IDUs. Yet their connection within the AIDS social movement has been weak. Meanwhile, these organizations had modestly better connections with the international platform, especially more recently. Their primary source of financial support is international donors (mainly bilateral projects – in particular, IHPCP/AusAID and FHI/USAID), with the exception of a few organizations with diversified donors and partners. Interestingly, their activities have not put much emphasize on activism, and have not exhibited much responsibility in fighting for the rights of drug users (e.g., guaranteed access to ARV, better laws, better policies and other basic issues of global human rights NGOs). Such advocacy is being promoted by IHPCP and more recently by the Open Society Institute as well.

Thus, despite the growing commitment by all players especially in recent years, all are convinced that the response to the HIV/AIDS epidemic so far is insufficient for the size of the problem. The dominance of NGOs has proved ineffective in scaling up efforts of AIDS services, particularly for IDUs.

In response to the problem, IHPCP's latest commitment in harm reduction has been to include the public health system in the service of AIDS to drug users and the empowerment of drug users as Indonesian citizens for universal access to health care.

## II- Description of the response so far and the role of IHPCP

The Indonesian response to the HIV/AIDS crisis among people who inject drugs is still modest. There is a clear consensus among stakeholders of an urgent need to scale up the response to the epidemic. In total, 41 NGOs are working in the field of harm reduction. Among these, 16 are conducting needle and syringe program projects, targeting 4,500 people who inject drugs on a monthly basis, all but one of these 16 NGOs supported by IHPCP. The other 25 organizations started modest syringe distribution after the second semester of 2006 with funding from the Partnership and the Global Fund, and they are partners of Family Health International in Indonesia. Besides NGOs, public health centres (Puskesmas) are also conducting harm reduction activities, including needle and syringe exchange. In July 2005 only one Puskesmas from Jakarta was developing harm reduction activities in Indonesia. By 2006, this had increased to 65. IHPCP and the local AIDS commissions are sharing the cost of these facilities for one year, with the commitment that future costs will be fully borne by the government. In September 2006, the City of Bandung Public Health Department in West Java, with their own funds, opened another 9 NSP in Public Health Centres. IHPCP provided technical support for planning and staff capacity building. So the current total of NSP slots in Indonesia by December of 2006 is actually 115.

These public health centres are targeting to reach another 23,000 people who inject drugs. The interaction of public health services and non-governmental organizations is the key element of interventions to scale up the response in the country. The role of the Public Health Centres, especially in the capital region of Jakarta and West Java (two of the main provinces of Indonesia) is to lead the response and use the infrastructure of the health system to scale the response to the level of the epidemic. The expansion was based on a successful experience conducted in the City of Sao Paulo, Brazil, from 2001 to the present [10]. Today the aim of the current projects is to achieve treatment of 30% of the injecting drug users in the country but because most efforts are new projects, the coverage is approximately 10% of the target. The scale-up proposed by KPA aims to achieve 70% of IDUs by 2010.

At the beginning of 2005 (after almost 7 years of the first NEP in Indonesia), most of the NEPs were still focused on the distribution/exchange of syringes only. Our effort after 2005 was to change the intervention for a comprehensive prevention package which includes, besides the sterile syringes, condoms, alcohol swabs, IEC (information, education, communication) material; projects conducted mainly on an outreach basis with a strong connection to the health system for referral in basic health care, drug treatment (highlighting methadone), and support and treatment for drug users at risk for HIV/AIDS.

Drug treatment in Indonesia is primarily based on drug free clinics for detoxification and rehabilitation, normally conducted by mental hospitals, NGOs or therapeutic communities. There is no official compulsory treatment in Indonesia. Buprenorphine is still expensive and not widely available. So far, approximately 300 doctors (mostly private doctors) across the country are certified to prescribe Buprenorphine. As well, anecdotal reports from IDUs in several provinces including Bali, West Java and other regions indicate a high rate of injecting Buprenorphine as heroin becomes scarcer in the market. Methadone was established first in Indonesia in 2003 by WHO and the Ministry of Health in two pilot projects, one in Jakarta and one in Bali. These two pilots together existed until the end of 2005, serving a population of approximately 300 drug users. Since 2004, IHPC has supported the main expenses of these two projects. Under the political influence of BNN in June 2005 (during the Anti-Drug World Day), Indonesian President Suscilo Bambang Yudoyono visited one of the clinics and announced a public program to expand methadone use based on its success so far. The expansion of methadone really started in 2006. By the end of 2006 there were 7 clinics serving approximately 1,000 clients. KPA's plan is to increase the number of drug users treated to more than 50,000 by 2010.

The work in prison is another front of harm reduction work in Indonesia. In June 2005, the Ministry of Justice and Human Rights launched the National Strategy for Prevention and Control of HIV/AIDS and Drug Abuse in Indonesian Correction and Detention Centres, for the period 2005–2009 [11]. The document detailing this program, the first of its kind in Asia, provides the framework for the work of prevention, care, support, and treatment of the HIV/AIDS epidemic inside the prison system. It was constructed with intensive input from IHPCP and other donors as well. Currently, only a few of the 396 prisons in Indonesia provide CST and HIV prevention; however some potentially effective demonstration projects are ongoing. The gold standard is the Balinese prison of Kerobokan where distribution of bleach and condoms for prisoners, as well as treatment with methadone and ARV are made available [12]. The central issue on the prison response to HIV/AIDS epidemic is the urgent need of increasing these interventions to address the sizeable problem. KPA's strategic plan is to cover 95 prisons by 2010, 20 of them with comprehensive programs like the one in Bali.

The legal basis for the Indonesian Response to HIV/AIDS among people who inject drugs is for the most part based on policy. Legislation in Indonesia is under debate to allow programs to assist in controlling the epidemic. There is no law against harm reduction in Indonesia, but prejudicial interpretation and misinterpretation of the current laws (all in effect before the HIV/AIDS epidemic) have resulted in many constraints, primarily in the realm of prevention. The Sentani Commitment signed in January of 2004 by the Head of the National AIDS Commission and many other authorities in Indonesia – and re-edited clearly delineating needle and syringe programs, as well as methadone programs – in June of 2005 is the main document supporting harm reduction activities in the country [13]. Memorandums of Understanding signed between ministers are also important support documents, such as those signed by the National AIDS Commission and the National Bureau on Narcotics. Public statements from authorities, including the President and the Vice-President of Indonesia, clearly supported harm reduction programs as well. Local authorities, such as the Vice-Governors of DKI Jakarta, West Java and Bali, but not limited to these officials, are publicly also supportive of harm reduction, including the commitment of their provinces' budgets to support the scaling up of the response. Some political resistance has arisen from some sectors of the police that prefer to maintain a focus on law enforcement, even though this strategy has previously been shown to fail. Some religious leaders are more resistant to the promotion of safe sex than to the promotion of safer use of drugs.

Advocacy of the police is the most difficult part of the job. Indonesia has a history of militarization of the street police that is still currently in effect. Police officers are underpaid, under-trained and under-equipped in Indonesia. As in many other countries, the police are susceptible to corruption and the use of unnecessary force. Politicized and influential, positions often change and sometimes all expenditures related to a specific advocate decrease or even disappear as a result of constant changes and are subsequently re-introduced. This can make for noticeable cost inefficiency.

The concept of universal access to AIDS treatment is new to Indonesia. The policy of free and universal access for ARV was implemented in 2004. According to the 3 × 5 initiative of WHO, Indonesia was recorded as having 10,000 people with AIDS (in need of ARV) by the end of 2005, of which 4,000, or 40% of the target, had been treated with ARV.

In Indonesia, national production of ARV is done by Kimia Farma, an Indonesian Governmental Pharmaceutical Company contracted by the Ministry of Health. First line medications produced in the country are Zidovudine, Nevirapine and Lamivudine. Indonesia has also made available other ARVs by import: Efavirenz; Stavudine and lopinavir + ritonavir - Kaletra [14] and gradually is increasing the choices. ARV is free of charge in the universal access spirit since the end of 2004; however ARV free of charge does not mean easy and free access. A CD4 account is still paid by the client with a cost of around US$ 13.00, an expensive blood test for Indonesians. Doctors still charge for the cost of consultation. It should be noted that about 20% of Indonesians are subsidized by the government based on poverty; thus, they obtain free health care, but 80% of the population still pays for health care.

A recent global review estimates that in Indonesia, people who inject drugs are about 31% of the people treated with ARV [15]. Thus, of the entire population of individuals who use injected drugs needing ARV treatment, about 25% are in treatment. This data takes into account equal likelihood for current or former injecting drug users. If we also consider the personal decisions of doctors who misunderstand the need for involving current injecting drug users in needed ARV treatment, this will likely worsen this scenario.

By 2006 IHPCP had attempted to stimulate among doctors in Indonesia the potential benefit of WHO and several other organizations to increase the number of current injecting drug users for ARV treatment [16]. From the previously mentioned 65 Public Health Centres are already actively engaged in NSP, 11 received training for implementation of VCT and ARV availability in community health centre settings. The joint initiative from IHPCP with the Indonesian Association of Doctors working with AIDS (PDPAI – Perhimpunan Dokter Peduli AIDS Indonesia) is also helping to promote the education of doctors in the country for universal access.

Formally, Indonesia is the only country in Asia that does not restrict people who inject drugs (including current users) from access to ARV treatment, and it is one of the few countries that produce the first line of ARVs for its own consumption. The KPA strategic plan has the provision to extend care, support and treatment of people who inject drugs to a total of 75 Public Health Centres (Puskesmas) by 2010, doubling the current possibilities for access.

Drug user participation is also currently a key element of the growing Indonesian response to the epidemic. Besides many NGOs made up of current and former drug users, two networks highlight the key participation of drug users. Jangkar is network of organizations working in the field of AIDS, and IDUSA is a Drug Users Individual Network. Both are obtaining strong support for their activities from IHPCP and other partners and are gradually being included in all important governmental meetings and decisions. Their agenda includes both the controlling of the HIV/AIDS epidemic and the key issue of the human rights of drug users.

The current scenario seems challenging. But realizing that as recently as two to three years earlier the current infrastructure for HIV/AIDS treatment was not in place, it's fair to say that **currently, all the components for a comprehensive response are in place in Indonesia**. The remaining question is how to expand this scenario, simultaneously guaranteeing the quality of interventions.

## III- Discussion and conclusion

Indonesia, the third biggest country in Asia, is facing an explosive epidemic driven by people who inject drugs. Even in a very inhospitable political and social environment, Indonesia is building a comprehensive response spearheaded by the commitment of the Indonesian government, province governments, civil society and international agencies. The response among people who inject drugs is being included in the public health system as a key strategy to push for the needed expansion of services. The role of the local governments is crucial, including their political and budget commitments, as a strong step in the sustainability of the response. The clear direction of the key interventions to address the HIV/AIDS epidemic that has affected Indonesia for the last 25 years is another important result. The clear focus on NSP, methadone, and care, support and treatment of people who inject drugs speaks to what needs to be done to address the epidemic. Initiatives from Indonesia such as the program to supply methadone inside prisons, and the promotion of ARV for current injecting drug users, are being perceived as the gold standard for all of Asia, a continent severely impacted by the HIV/AIDS epidemic. There is a long way to go in Indonesia to significantly impact the epidemic and thus celebrate the saving of thousands of lives, but the bases are very well established.

As UNAIDS head Peter Piot stated: "...we need to do more of the wonderful things we have been doing so far".

## References

[B1] UNAIDS (2006). Global Report of the HIV/AIDS Epidemic, Geneva.

[B2] Monitoring the AIDS Pandemic (MAP) (2004). AIDS in Asia: Face the facts, Geneva.

[B3] Ministry of Health of Indonesia (2006). Report on HIV/AIDS cases to September of 2006, Jakarta.

[B4] Ministry of Health of Indonesia (2006). Estimate of the People Living with HIV/AIDS, released on December 1, Jakarta.

[B5] Global Fund to fight AIDS, Tuberculosis and Malaria, report from 2006. http://www.theglobalfund.org.

[B6] National Narcotics Board and Center of Health Research Universitas Indonesia (2004). A Study on the social and economic cost of the abuse of drugs in 10 major cities in Indonesia, Jakarta.

[B7] National Narcotic Board. Republic of Indonesia (2005). Annual Report 2005, Jakarta.

[B8] Australian National Council on Drugs (2006). Situational Analysis of Illicit Drug Issues and Responses in the Asia-Pacific Region. A Burnet Institute and Turning Point Alcohol and Drug Centre collaborative study.

[B9] Setiawan Made, Patten Jane, Triadi Agus, Yulianto Steve, Terryl Adrnyana, Arif Moh (1999). Report on injecting drug use in Bali (Denpasar and Kuta): results of an interview survey. International Journal on Drug Policy.

[B10] Bueno Regina, Trigueiros Daniela, Fabio Mesquita, Celia Regina de Souza (2003). *El Proyecto de Reduccion de Danos de la Ciudad de Sao Paulo*. ETS/SIDA, La Nueva Cara de la Lucha Contra la Epidemia en la Ciudad de Sao Paulo.

[B11] Winarso Inang, Irawati Ira, Eka Bambang, Nevendorff Laura, Handoyo Patri, Salim Hendra, Mesquita Fabio (2006). *Indonesian national strategy for HIV/AIDS control in prisons: a public health approach for prisoners*. International Journal of Prisoner Health.

[B12] Irawati Ingrid, Mesquita Fabio, Winarso Inang, Hartawan, Asih Putu (2006). *Indonesia Sets Up Prison Methadone Maintenance Treatment*. Addiction (News and Notes).

[B13] Sentani Commitment, National AIDS Commission of Indonesia (KPA) http://www.papuaweb.org.

[B14] Ministry of Health of Indonesia (2004). National Guidelines on Antiretroviral Therapy – "Pedoman Nasional Terapi Antiretroviral", Jakarta.

[B15] Aceijas Carmen, Oppenheimer Edna, Stimson Gerry, Ashcroft Richard E, Matic Srdan, Hickman Mattew, on behalf of the Reference Group on HIV/AIDS Prevention and Care among IDU in Developing and Transitional Countries (2006). Antiretroviral treatment for injecting drug users in developing and transitional countries 1 year before the end of the "Treating 3 million by 2005. Making it happen. The WHO strategy ('3by5'). Addiction.

[B16] World Health Organization Clinical Protocol on HIV/AIDS Treatment and Care for Injecting Drug Users. http://www.euro.who.int/aids/treatment/20060801_1.

